# Phase I oncology trials incorporating patient choice of dose

**DOI:** 10.1038/bjc.2012.378

**Published:** 2012-08-28

**Authors:** L W Huson

**Affiliations:** 1 Division of Experimental Medicine, Imperial College London, Hammersmith Campus, Du Cane Road, London W12 0NN, UK

**Keywords:** phase I, oncology, continual reassessment method, ethics, dose escalation

## Abstract

**Background:**

Patients recruited in phase I oncology trials are often treated at doses lower than the maximum tolerated dose (MTD), and therefore may not receive the most efficacious dose available, despite their expectations to the contrary. This report investigates the consequences of allowing a patient choice of dose within a common dose-escalation scheme.

**Methods:**

Trials using the continual reassessment method of dose escalation are simulated, with a modification of the rules to allow patients to choose a higher dose if they wish. The effect of allowing this choice is assessed in terms of probability of toxicity and probability of being treated at the MTD or higher.

**Results:**

The simulations show that allowing a patient choice of dose reduces the proportions of patients treated at doses lower than the MTD, and has little impact on the overall probability of correct identification of the MTD.

**Conclusion:**

The results illustrate the principle that a choice of dose can be offered to patients in such trials without compromising the overall properties of the study.

During the development of novel anticancer drugs, phase I trials are conducted with the objective of identifying the maximum tolerated dose (MTD) – the maximum dose that can be administered without an unacceptably high risk of toxicity. Patients recruited into such trials are often those who have exhausted all standard treatment options, and an experimental drug is the only possible therapeutic option for many of them. A key feature of these phase I trials is that the dose-escalation schemes typically begin with low doses. These are not only believed to be associated with minimal risk of toxicity but are also expected to be less efficacious than the higher doses that may be used later in the trial. Studies have shown, however, that a majority of patients, when they agree to participate in phase I oncology trials, do so in the hope that they might receive some benefit from the test treatment, and there is evidence that patients are willing to accept a greater risk of toxicity from experimental therapies (Merepol *et al*, 2003; Nurgat *et al*, 2005; Brunetto *et al*, 2011).

Rosa *et al* (2006) report the case of a woman with ovarian cancer who was offered the chance to be the first patient, treated at the lowest dose, in a phase I trial. The patient was informed that the first dose level was lower than that which was expected to be selected as the MTD, and being concerned about not receiving a potentially more efficacious dose, she asked if she could delay entry to the study until a later time when higher doses might be available. Patients are rarely allowed to choose their doses in phase I oncology trials, and allowing patients to delay entry to receive a higher dose would undoubtedly affect the logistics of the trial. An alternative, therefore, might be to permit patients to choose, within limits, which dose they would like to receive, as long as this did not affect the overall properties of the trial. This report documents simulation studies showing the impact that a patient choice of doses would have on the performance of a widely used Bayesian model-based dose-escalation scheme – the continual reassessment method (CRM) (O’Quigley *et al*, 1990).

## The continual reassessment method

Detailed descriptions of the CRM are available in the literature (Garret-Mayer, 2006; Ishizuka and Morita, 2006). Implementation requires a choice of a set of doses, a statistical model to describe the relationship between the probability of toxicity and the dose, a set of prior probabilities for toxicity at each dose, and a target ‘acceptable’ toxicity probability. The first patient recruited is treated at the starting dose, and based on the outcome, the dose–response model is updated to give a recommendation for the next dose. Successive patients are treated at doses recommended by the updated model until the trial stops, typically after a pre-defined maximum number of patients.

In the original presentation of the CRM, the authors reported simulations showing the probability with which the algorithm correctly identifies the MTD, and the percentage of patients who experience toxicity. For example, the first scenario in the original paper is a trial testing six dose levels, with prior toxicity probabilities of 0.05, 0.1, 0.2, 0.3, 0.5 and 0.7 respectively, and a target toxicity probability of 0.20. In this scenario, the CRM correctly identifies the MTD with probability of approximately 0.45, and 22% of patients would be expected to experience toxicities. However, these performance statistics depend on the choice of the prior toxicity probabilities, and the properties of the method should be studied for the particular circumstances of each individual phase I trial.

## Simulation study of the CRM incorporating patient choice of dose

To illustrate the principle of using the CRM with patient choice of doses, the simulations reported here use the typical phase I trial scenario taken from the original CRM paper. The simulated trial uses six dose levels with prior toxicity probabilities noted above. A fixed sample size of 20 patients is used, with target toxicity probability of 0.20, and the toxicity response model is the single parameter logistic. The first dose administered in each simulated trial is dose 3, which has prior toxicity probability equal to the target. In the first series of simulations, a proportion of patients, ranging from 0 to 100%, is allowed to choose to receive a dose one level higher than the current MTD recommended by the Bayesian model. For each case, 2000 simulations were run. The results are shown in Table 1.

The simulations with no patients allowed to choose a higher dose reveal the same results as in the original CRM paper. For these prior toxicity probabilities, the correct MTD is identified with probability 0.45, and 21.5% of patients, on average across the simulated trials, experienced a toxicity. Also shown are the percentages of patients dosed at the MTD and the percentage who receive doses lower than the MTD. As more patients opt to receive higher doses, the probability of correct identification of the MTD declines slightly, but this reduction is of no practical significance. The proportion of patients experiencing a toxicity increases, to a level consistent with the toxicity probability associated with the dose one level higher than the MTD. However, the number of toxicities does not increase among the patients who choose not to receive a higher dose. The proportion of patients treated at the MTD declines as more patients opt for a higher dose, and the proportion of patients treated at doses lower than the MTD, that is, at less efficacious doses, also declines. Overall, the results suggest that allowing patients this simple choice does not markedly affect the performance of the CRM for this set of prior toxicity probabilities, and affects only the probability of a toxicity. Similar results are seen if patients are allowed to choose *any* of the doses higher than the one currently identified as the MTD (Table 2).

The conclusion from these simulations is that the only impact of these patient choice regimes is that patients who choose to receive a higher dose are more likely to experience a toxicity, but that the overall properties of the CRM are not significantly impacted. Under these patient choice regimes, fewer patients are treated at doses lower than the MTD, and there is therefore a possibility that patients may experience greater efficacy, *albeit* at the expense of a higher risk of toxicity. Allowing such a choice may therefore be an option that could be considered, at least in some phase I oncology trials. It should be stressed, however, that before such a regime is contemplated, simulations should be performed to determine the properties of the method under the particular conditions expected for the specific trial being planned. For this purpose, the codes used in this study are available from the author.

## Discussion

This study demonstrates the principle that allowing patients in phase I oncology trials a choice of dose need not affect the overall properties of the dose-escalation procedure. The CRM was selected to demonstrate this, not because it is necessarily the best escalation algorithm but because it permits measurement of numerical criteria that allow overall trial performance to be assessed. Other dose-escalation schemes are available within which patient choice of dose might be permitted. The only example of allowing patients to choose doses in this way is reported by Daugherty *et al* (1998), who offered patients within their trial a choice of dose, within certain limits, and reported that 76% of patients exercised this option and 28% selected the highest dose.

Phase I oncology trials have been discussed extensively in the literature and there is still no consensus about their ethical aspects (Agrawal and Emanuel, 2003). The issue driving the debate is the fact that these trials recruit patients who have few treatment options remaining. Patients are recruited into trials in which they are expected to receive little benefit, and in which they risk toxic effects. Attempts have been made to quantify these risks and benefits (Joffe and Miller, 2006), and the figures suggest that no more than 5% of patients experience efficacy in phase I cancer studies. Although some newer agents have achieved better efficacy, a recent review suggests no efficacy difference overall between targeted biologics and cytotoxic drugs (Roberts *et al*, 2004). Overall, it is hard to disagree with Rosa *et al* who say: ‘*given the choice between an almost certainly sub-therapeutic dose versus a dose that could be more likely to be therapeutic, but also more likely to be toxic, it is possible that the majority of patients would prefer the dose more likely to be therapeutic. If they do have this preference … it is difficult to see why they should not be offered the dose more likely to benefit them*.’ The objective of this report is not to advocate that all patients should be given a high dose or that there should be no attempt to determine the MTD, but to suggest that there is an ethical argument in favour of allowing patient choice. Greater patient autonomy might in itself be an adequate ethical justification for allowing this choice. The option to choose a higher dose would require extensive discussion between individual patients and their treating physicians, and should be made in the context of a detailed assessment of benefits and risks.

## Figures and Tables

**Table 1:** Performance characteristics of the CRM allowing patients to choose one dose increment

**Percentage of patients opting for higher dose (%)**	**Percentage of simulated trials correctly identifying MTD (%)**	**Average percentage of patients experiencing toxicity (%)**	**Average percentage toxicity in patients not choosing higher dose (%)**	**Average percentage of patients dosed at correct MTD (%)**	**Average percentage of patients dosed at less than correct MTD (%)**
0	44.9	21.5	21.5	33.7	32.0
10	44.7	22.8	21.5	32.6	31.4
20	43.0	24.1	21.7	31.1	27.2
30	44.0	25.3	21.6	30.2	24.6
40	43.7	26.7	21.3	28.9	23.6
50	44.5	27.8	21.4	27.5	21.2
60	42.2	29.3	21.5	26.8	18.4
70	44.2	30.2	21.6	24.9	16.4
80	41.9	31.8	21.2	24.0	14.5
90	43.2	32.6	21.6	22.5	12.5
100	42.6	34.0	—	21.7	10.7

**Table 2:** Performance of the CRM when patients are allowed to choose any dose higher than the current recommended MTD

**Percentage of patients opting for higher dose (%)**	**Percentage of simulations correctly identifying MTD (%)**	**Average percentage of patients experiencing toxicity (%)**	**Average percentage toxicity in patients not choosing higher dose (%)**	**Average percentage of patients dosed at correct MTD (%)**	**Average percentage of patients dosed at less than correct MTD (%)**
0	44.5	21.7	21.7	33.0	32.7
10	43.8	23.1	21.6	32.3	30.3
20	44.4	24.3	21.3	31.1	28.1
30	43.7	26.2	21.5	30.5	25.6
40	42.6	27.4	21.5	29.0	23.2
50	43.0	29.0	21.5	27.8	21.7
60	43.0	30.5	20.8	26.5	19.7
70	42.3	32.1	22.0	25.7	17.6
80	41.9	33.7	21.3	24.4	15.4
90	39.8	35.0	20.6	23.1	13.1
100	42.8	36.7	—	22.3	11.7
